# Proteome of the phytopathogen *Xanthomonas citri *subsp. *citri*: a global expression profile

**DOI:** 10.1186/1477-5956-8-55

**Published:** 2010-11-09

**Authors:** Márcia R Soares, Agda P Facincani, Rafael M Ferreira, Leandro M Moreira, Julio CF de Oliveira, Jesus A Ferro, Maria IT Ferro, Rogério Meneghini, Fábio C Gozzo

**Affiliations:** 1 Laboratório Nacional de Luz Sincrotron (LNLS), Campinas, SP, Brazil; 2 Departamento de Bioquímica, Instituto de Química, Universidade Federal do Rio de Janeiro (UFRJ), Rio de Janeiro, RJ, Brazil; 3 Departamento de Tecnologia, Faculdade de Ciências Agrárias e Veterinárias de Jaboticabal, Universidade Estadual Paulista (UNESP), SP, Brazil; 4 Departamento de Ciências Biológicas, Instituto de Ciências Exatas e Biológicas, Universidade Federal de Ouro Preto, Ouro Preto, MG, Brazil; 5 Núcleo de Pesquisas em Ciências Biológicas (NUPEB), Universidade Federal de Ouro Preto, Ouro Preto, MG, Brazil; 6 Departamento de Ciências Biológicas, Universidade Federal de São Paulo (UNIFESP), Diadema, SP, Brazil; 7 Fundação de Apoio a Universidade Federal de São Paulo, São Paulo, SP, Brazil; 8 Instituto de Química, Universidade Estadual de Campinas, UNICAMP, Campinas, SP, Brazil

## Abstract

**Background:**

Citrus canker is a disease caused by *Xantomonas citri *subsp.*citri (Xac)*, and has emerged as one of the major threats to the worldwide citrus crop because it affects all commercial citrus varieties, decreases the production and quality of the fruits and can spread rapidly in citrus growing areas. In this work, the first proteome of *Xac *was analyzed using two methodologies, two-dimensional liquid chromatography (2D LC) and tandem mass spectrometry (MS/MS).

**Results:**

In order to gain insight into the metabolism of *Xac*, cells were grown on two different media (**NB **- Nutrient Broth and **TSE **- Tryptone Sucrose broth enriched with glutamic acid), and proteins were proteolyzed with trypsin and examined by 2D LC-MS/MS. Approximately 39% of all predicted proteins by annotation of *Xac *were identified with their component peptides unambiguously assigned to tandem mass spectra. The proteins, about 1,100, were distributed in all annotated functional categories.

**Conclusions:**

This is the first proteomic reference map for the most aggressive strain of *Xanthomonas *pathogen of all orange varieties. The compilation of metabolic pathways involved with bacterial growth showed that *Xac *expresses a complete central and intermediary metabolism, replication, transcription and translation machineries and regulation factors, distinct membrane transporters (ABC, MFS and pumps) and receptors (MCP, TonB dependent and metabolites acquisition), two-component systems (sensor and regulatory components) and response regulators. These data corroborate the growth curve *in vitro *and are the first reports indicating that many of these genome annotated genes are translated into operative in *Xac*. This proteomic analysis also provided information regarding the influence of culture medium on growth and protein expression of *Xac*.

## Background

Advances in genome sequencing have resulted in the description of a huge number of genes; some of these genes are hypothetical. However, the functions of many of these genes and the relationship among the many proteins that constitute the proteome of a cell or tissue cannot be easily determined. In addition, about half of the predicted proteins have no inferable functions, especially in microorganisms [1,2].

Many approaches, such as two-hybrid systems and proteomics, have been used to describe genes that are effectively translated into proteins for systems of interest without considering transcriptomics or other methodologies that focus on RNA expression. Although they are complementary techniques, proteomics has the advantage of looking directly at the protein level, as gene and protein levels are not always equivalent due to processing and regulation of gene expression at both the transcriptional and translational levels [3].

Despite the large number of proteomic studies, particularly for bacteria, there are few reports about the genus *Xanthomonas*. *Xanthomonas *consists of mainly phytopathogenic bacteria that infect a wide variety of economically important plants, such as brassicas, rice, citrus, vine, cassava, pepper, and tomato. Most proteomic analyses involving organisms of the Xanthomonadaceae family have studied *Xanthomonas campestris *pv. campestris [4-7], due to the completion of its genome sequencing in 2002 [8] and its ability to infect *Arabidopsis thaliana*, a model plant for genetic studies [9].

*Xanthomonas citri *subsp. *citri *(*Xac *= *Xanthomonas axonopodis *pv. *citri*) [10,11], the causal agent of citrus canker, a disease that affects most commercial citrus cultivars and causes extensive damage to citriculture around the world [12,13], is also a good target for proteomic studies. In addition, its complete genome has been sequenced [8] and compared with those from other strains [14-16].

Few studies have focused on *Xac *proteins, but instead have examined specific genes involved in pathogenicity and adaptation mechanisms [17,18].

This work reports our analysis of the global protein expression profile of *Xac *using two distinct conditions of *in vitro *growth. First, we used a rich medium, Nutrient Broth, which is usually used for the cultivation of microorganisms. We also used Tryptone Sucrose broth, TSE, enriched with glutamic acid. TSE is a version of TSA medium [19] without agar and was chosen due to the high growth potential of Xanthomonas in this medium compared to NB medium. The two compounds in TSE medium, sucrose and glutamic acid, were individually determined as being fundamental to the growth process and are also considered important in mimicking the plant environment [20,21].

We aimed to compare the proteome of Xanthomonas grown in NB medium as opposed to TSE medium to understand the differences in the profile of the growth curve. We verified that the presence of sucrose and glutamic acid induced for the expression of genes involved directly or indirectly with pathogenicity.

These two proteomes were analyzed by two-dimensional liquid chromatography-tandem mass spectrometry (2D-LC-MS/MS) to improve protein coverage [22-24].

## Results

### Proteomic analysis from *Xac *under two distinct conditions

**Figure 1:**
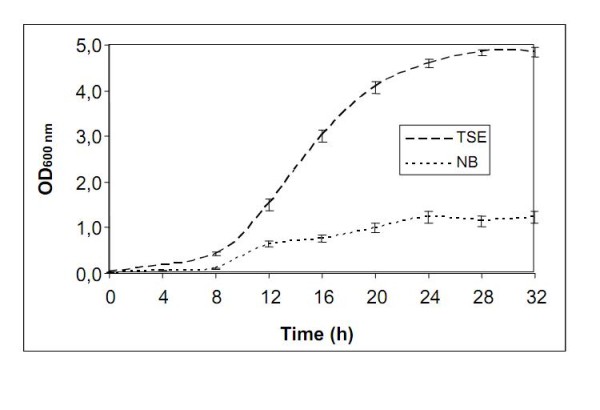
***G*rowth curve profiles of *Xanthomonas citri *subsp. *citri *in TSE and NB media**. Bacteria were grown at 28°C in Nutrient Broth (NB - 5 g/L peptone and 3 g/L meat extract) or Tryptone Sucrose Broth (TSE - 10 g/L tryptone, 10 g/L sucrose and 1 g/L sodium glutamate) media with shaking at 200 rpm for 16 hours.

The *Xanthomonas citri *subsp. *citri *was grown at 28°C in NB or TSE media. The cells were collected at an optical density (OD_600_) corresponding to log phase (Figure 1). The protein extracts were proteolyzed, and the tryptic peptides were separated into 100 fractions in the first dimension (see Additional file 1), followed by nLC-MS/MS investigation. Approximately 119,000 MS/MS peptide spectra for NB and 100,000 MS/MS peptide spectra for TSE were collected and analyzed. The MS/MS spectra led to the identification of 1,162 proteins from bacteria grown in NB and 1,261 proteins from bacteria grown in TSE (see Additional file 2).

### Data validation

The reversed sequence database was used as a filter for incorrect or ambiguous peptides. Using the same (Methods Section) parameters for the *Xac *database, the search of all of the MS/MS spectra returned 5% false positive identifications on the reversed sequence databank, showing good reliability for the method.

### Proteome × Genome

**Figure 2:**
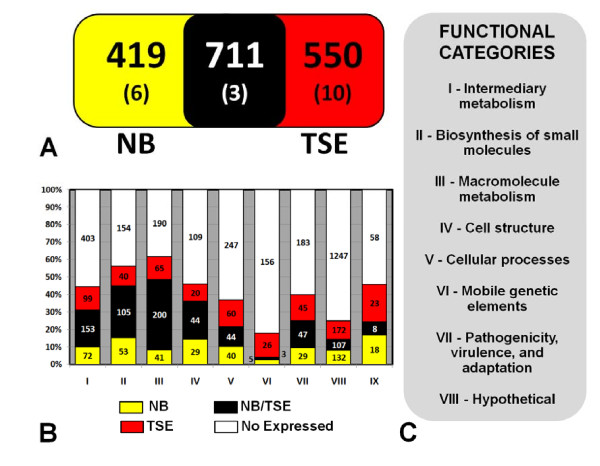
**Proteomic analysis of *Xanthomonas citri *subsp. *citri *under non-infecting conditions**. **(A) **A comparison of identified proteins from *Xac *grown on NB culture medium and *Xac *grown on TSE culture medium (* denotes plasmid's protein). **(B) **Comparison of the *Xac *proteins identified in this study and the genomic prediction per categories **(C)**. For clarity, proteins with undefined functions are excluded from the chart. The red color represents *Xac *proteome in TSE, yellow color represents *Xac *proteome in NB, black represents protein detected in both media and white refers to proteins not detected.

This approach provided high protein coverage, and the results were compatible with those obtained from genome analysis (see Additional file 3 and Figure 2). The total number of proteins found in the present study (1,702) corresponds to almost 40% of all of the proteins predicted by the genome, which is equivalent to that of *E. coli*, the most comprehensive proteome [25]. The identified *Xac *proteins were classified according to their annotated function (Table 1). A majority of the proteins were classified as hypothetical proteins (413) or related to metabolism (830): categories I (intermediary metabolism), II (metabolism of small molecules) and III (macromolecule metabolism).

**Table 1:** Comparison of genome with proteomic analysis from *Xanthomonas citri *subsp. *citri*.

Categories		Genome	Proteome	Prot/Gen %
I	Intermediary metabolism	727	324	45
II	Biosynthesis of small molecules	352	198	56
III	Macromolecule metabolism	496	308	62
IV	Cell structure	202	95	48
V	Cellular processes	391	145	37
VI	Mobile genetic elements	190	44	23
VII	Pathogenicity, virulence and adaptation	304	125	41
VIII	Hypothetical	1658	413	25
IX	ORFs with undefined category	107	49	46

	**Total**	**4427**	**1702**	**39**

The number of proteins detected in this study independent of growth conditions is compared with the total expected by the genome

#### Intermediary and small molecule metabolism

**Figure 3:**
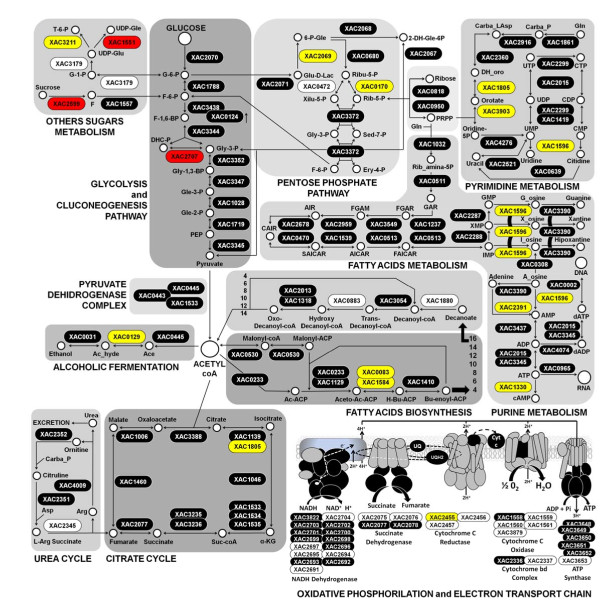
**The protein expression profile of *Xanthomonas citri *subsp. *citri *according to energy production**. The pathways illustrated here are almost complete. The expressed proteins in TSE are represented in red boxes, the expressed proteins in NB are represented in yellow boxes, black boxes represent proteins detected in both media and white boxes represent non-expressed or non-detected proteins.

The *Xac *genome contains 727 and 352 predicted genes classified in intermediary metabolism and biosynthesis of small molecule categories, respectively [8]. We identified 324 proteins assigned to the intermediary metabolism category, 106 of which are related directly to energy metabolism (51%, 106 out of 209 in the genome), involving the complete glycolysis/gluconeogenesis, citrate cycle (TCA cycle), pentose phosphate pathway, other sugar metabolism, urea cycle, pyrimidine and purine biosynthesis, and fatty acid synthesis and degradation pathways (Figure 3). We also observed 23 response regulators (sensor, regulator and hybrid-proteins), 9 complete and 13 incomplete two-component systems, all proteins required for polyamine biosynthesis (XAC0484, XAC3924 and XAC3923) and 42 proteins involved in nucleotide biosynthesis (out of 51 predicted by genome).

#### Macromolecule metabolism

**Figure 4:**
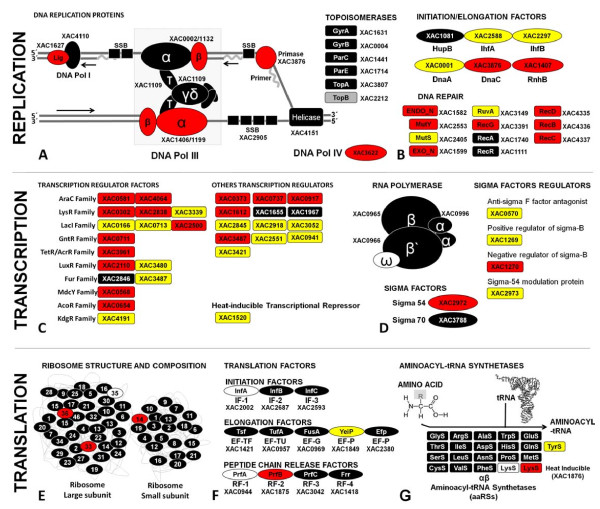
**Distribution of identified proteins of *Xanthomonas citri *subsp. *citri *involved in replication, transcription and translation process**. In total, 163 proteins (9.6% of the proteome) are involved in these processes. The expressed proteins in TSE are represented in red boxes, the expressed proteins in NB are represented in yellow boxes, black boxes represent proteins detected in both media and white boxes represent non-expressed or non-detected proteins.

We detected 308 of the 496 proteins (62%) predicted by the genome to be involved in macromolecule metabolism. Several of these expressed proteins are involved in the metabolism of DNA (53%, 71 out of 135), RNA (53%, 110 out of 209) or protein (66%, 103 out of 157) and include initiation, elongation, transcription, sigma and translation factors (Figure 4). In addition, ribosomal proteins as well as aminoacyl-tRNA synthetases to all tRNA were also expressed, consistent with the expected number of genes (54 ribosomal proteins = genome) (Figure 4E). These processes show the potential for *Xac *replication, in accordance with its growth curve profile (Figure 1).

#### Cell structure

**Figure 5:**
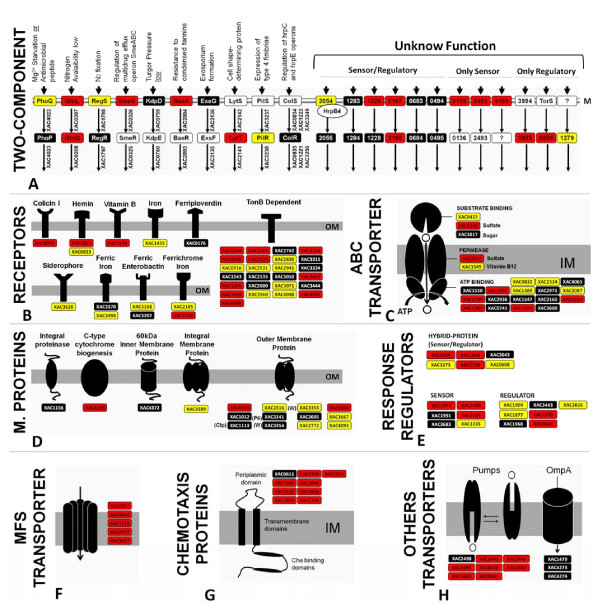
**Distribution of identified proteins of *Xanthomonas citri *subsp. *citri *related to membrane, transport and gene regulation**. In total, 154 proteins (9.1% of the proteome) are involved in these processes. The expressed proteins in TSE are represented in red boxes, the expressed proteins in NB are represented in yellow boxes, black boxes represent proteins detected in both media and white boxes represent non-expressed or non-detected proteins.

Similar to other bacterial proteomes, a large number of proteins involved in cell structure and cellular processes, such as surface proteins, were detected in the *Xac *proteome. In particular, we identified proteins involved in pilus synthesis, corresponding to genes XAC3098-3102 (the complete cluster) and subunits pilB, A, C, Y1 and X from distinct clusters. Among the members of this category, we also found 12 distinct outer membrane proteins, 5 pumps and 3 OmpA proteins (Figure 5).

#### Cellular processes

In this study, 145 proteins of this category were found; among them, 17 are related to cell division (66%, 17 out of 27 of genome), 105 are related to transport (37%, 105 out of 281) and 4 distinct proteins are related to osmotic processes (XAC0304, XAC0604, XAC2915, and XAC0888).

#### Mobile genetic elements

In this class, 44 proteins were found, of which 12 proteins have phage-related functions and are prophages, 12 have plasmid-related functions and 20 have transposon- and intron-related functions.

#### Pathogenicity, virulence and adaptation

Proteins involved in adaptation (63%, 15 out of 24 in the genome) were also identified, as well as proteins related to pathogenicity. Although *hrp *gene expression is generally induced in plants or under certain *in vitro *conditions [26], four protein constituents of the type III secretion system apparatus and two proteins related to hypersensitivity were found in the *Xac *proteome during growth in TSE medium: HrcN (XAC0412), HrpF (XAC0394), HrcU (XAC0406) and HrcV (XAC0405), XopAE (XAC0393) and a PthA isoform was found in the two protein extracts (NB medium). The expression of proteins for both type II (XAC3534/35 - D/N, XAC3537 - L and XAC3544 - E subunits) and type IV secretion systems (XAC2614-XAC2623) was also verified.

#### Expression of hypothetical genes

In an attempt to find potential functions for the hypothetical proteins expressed by *Xac*, the BLASTp program was used to search for sequence similarities [27]. A BLAST search for homologous proteins in *Xanthomonas campestris *pv. vesicatoria returned 209 proteins with identity > 34%, coverage > 49% and e-values less than or equal to 10^-19 ^(see Additional file 4). Examples of proteins previously proposed as hypothetical are XAC3997, XAC2966, XAC3898 and XAC1756, which now are categorized as proteins with putative functions, such as ABC transporter permease, BolA superfamily transcriptional regulator, membrane-bound metalloendopeptidase and PhoH-like protein, respectively.

#### ORFs with undefined category

A considerable number of proteins (49) without a specific category were identified in this work. These proteins were identified in both of the two treatments, suggesting that the proteins play an important role in metabolism. For example, the products of XAC2529 and XAC3245 (RhsD proteins) may have a putative function involved in pathogenesis or virulence due the cellular location. This protein was found in bacterial outer membrane or vesicles secreted together with proteins secreted by the Type III secretion system in other species of *Xanthomonas *[7]. Accordingly, Laia and coworkers [28] reported that a mutant for XAC3245 gene in *Xac *causes significant reduction of necrosis during the plant infection process.

## Discussion

In the present work, the proteomic analysis of *Xac *cultivated in non-infecting conditions revealed the presence of 1,702 proteins, corresponding to 39% of all of the proteins predicted by the genome. A good correlation with the genome was observed (Figure 2), except for the class of mobile genetic elements, which exhibits more intense activity under stress conditions [29,30]. In addition to regulation factors, proteins involved in the pathways of DNA replication, transcription and translation are associated with active growth.

*Xac *expresses almost every protein involved in energy metabolism pathways, such as the TCA cycle, pentose pathway, fermentation, biosynthesis and degradation of fatty acids, glycolysis/gluconeogenesis, and purine and pyrimidine biosynthesis (Figure 3). These observations might be explained by the localization of these proteins because the detected products are mainly cytosolic proteins. It is not surprising that a large proportion of the identified proteins belong to energy metabolism. Nonetheless, this work is the first large-scale analysis for an organism of the Xanthomonadaceae family.

The presence and detection of the transcription factors, sigma factors, ribosomal subunits, translation factors and aminoacyl-tRNA synthetases (Figure 4) illustrate the good agreement between the genome and proteome of *Xac *under the investigated conditions.

A large number of membrane constituents were detected, including 22 ABC transporters and other response regulators. In addition, 154 proteins related to these processes were found, corresponding to 9.1% of the proteome. In addition, nine sets of two-component systems were completely identified (sensor + regulator) (Figure 5A), with two related to nitrogen metabolism (XAC02907/0208 - GlnLG and XAC1798/1797 - RegSR) and the others (e.g., XAC2054 and XAC2055) with unknown function. Alegria and coworkers [31] described the interaction between XAC2054 and the product of the *hrpB *gene, which was also found in the proteome, suggesting an association with the type III secretion system. The TonB-dependent receptors (25 of 46 annotated), which participate in the uptake of iron or siderophores, were also highly expressed. The significant expression levels of these receptors and 13 more specific iron receptors demonstrate the ability of *Xac *to take up these compounds, which is fundamental to the growth of microorganisms, and some are related to pathogenicity [32].

*Xac *expressed a large number of components related to cell structure. These proteins play an important role during bacterial infection and host adhesion. Examples of proteins in this group include type IV fimbriae and pilus proteins, which are important to the processes of colonization and biofilm formation. The co-expression of hemagglutinin protein, which is coded by the gene XAC1815, corroborates this physiological condition, which is fundamental to pathogen adaptation in the plant tissue with consequent induction of pathogenicity [33]. In addition, proteins involved in different cellular processes were detected, including a large set of proteins related to cell division and a larger number of transporter and chemotaxis proteins (Figure 5). These proteins are mainly involved in adaptation to culture conditions.

Mobile genetic elements represented about 3% of the *Xac *proteome, a minor group of the detected proteins. This result was expected because these genes are generally related to survival response mechanisms, such as antibiotic resistance and virulence [34]. In this study, *Xac *was grown on privileged media under non-stressing conditions. The genes corresponding to the identified proteins, such as transposases (XAC1872 and XAC3233), were located mainly in the chromosome.

Additional experiments are needed to investigate the appropriate conditions for the expression of these genes, which have significant importance for genome plasticity and evolution [35].

The expression of genes related to pathogenicity was surprising, especially in TSE medium (Additional file 5). Among these proteins, members of secretory systems are the most investigated [36-39].

The type III secretion system is a generally conserved mechanism found in both plant and animal pathogens for the export, secretion and delivery of specific proteinaceous effector molecules (virulence or pathogenicity factors) directly or indirectly into host cells in a contact-dependent manner. The type II secretion system is involved with the secretion of cell wall-degrading enzymes, such as carbohydrate esterases and proteases.

Genes encoding components of the secretion apparatus are not constitutively expressed but activated *in planta *and in minimal media mimicking the environmental conditions present in the plant apoplast. Some proteins related to secretory systems were identified in this work; these proteins were detected only during bacterial growth priority on TSE medium. NB medium has been suggested as a more appropriate medium to compare the differential expression of proteins in infectious non-inducing conditions. Furthermore, a higher level of xanthan gum production has been observed in TSE culture (data not shown), which is associated with virulence factors [40]. Recently, new genes involved in the pathogenesis process have been reported [28], and the author showed that expression of these genes is dependent on the plant-pathogen interaction. Among these genes, the XAC0340 product was the only one detected in TSE media, which could be associated with the presence of specific components in this medium, such as sucrose or glutamic acid.

Sucrose has been described as an inducer of hrpF gene expression in *Xanthomonas campestri*s pv vesicatoria, in this case by modifying the culture medium MM1 with additional amino acids that have sulfur in their composition [20]. Glutamic acid has been reported to potentiate the growth of Xanthomonas, particularly in the case of *Xanthomonas fuscans *B, which is consistent with the growth curves. Quorum sensing due to the high density of cells would stimulate the expression of genes related to the type III secretion system, as described for enteropathogens [41,42] and phytopathogens [43,44]. These results support the idea that NB is a more appropriate medium for control experiments.

One goal of this study was to provide experimental annotation of predicted genes. The products of these genes may have important roles in bacterial development. A total of 209 of the 413 hypothetical proteins have homologs in *Xanthomonas campestris *pv. vesicatoria, which allowed us to hypothesize their function (see Additional file 4).

Finally, this proteomic analysis also provided information regarding the influence of culture medium on the growth and protein expression of *Xac*. Figure 1 shows the more effective growth in TSE medium, reflected in the larger number of proteins expressed in this condition. As shown in Figure 2A, a significant number of proteins were exclusively detected under each condition. These proteins probably depend directly on the components of each medium. Therefore, the choice of medium can be crucial in developing a control experiment.

## Conclusions

This report is the first presentation of a proteome map of the most aggressive strain of *Xanthomonas *pathogen of all orange varieties, *Xac *[16]. We used a 2D-LC-MS/MS strategy to characterize this proteome. Protein mixtures were digested with proteases, and the resulting peptides were separated by multidimensional liquid chromatography and analyzed sequentially by MS/MS, resulting in very high protein coverage.

We were able to identify proteins with a variety of functions covering most cellular processes, including regulatory and signal transduction proteins, even though these proteins are normally present in small amounts in the cell.

The complete set of energy metabolism pathways identified in this study denotes the ability of Xanthomonas to convert carbon sources from other biomolecules of fundamental importance for metabolism, as shown in Figure 3, and which helps to explain the profile of the growth curve (Figure 1). In addition to this metabolic energy profile, annotated genes related to metabolic function of macromolecules were determined in this study. Initiation factors for replication, transcription, and translation and sigma factors are shown in Figure 4. However, the receptors and membrane transporters identified in this study drew the most attention. The quantity and diversity of our results support the growth dynamics, colonization and spread of Xanthomonas in plant tissues. The two-component systems and response regulators, membrane receptors closely related to the uptake of iron and membrane transporters (MFS, ABC, OMPs and other pumps), are also interesting. Many of these proteins were annotated by sequence homology, but their functions have not been characterized in Xanthomonas, as shown in Figure 5.

This platform for proteomics, in corroboration with other tools for functional analysis of the genome of *Xac*, like transcriptomics [45], mutatomics [28] or two-hybrid systems [37,38], will enable a more integrated understanding of metabolic pathways, essential for the adaptation of pathogens in plant tissue. Additional studies directed toward the development of biotechnological tools to combat the pathogen may help to reduce worldwide crop losses due to the genus *Xanthomonas*.

## Methods

### Bacterial strains, growth and cell lysis

The *Xanthomonas citri *subsp. *citri *strain 306 used both in the genome project [8] and in this study was obtained from the culture collection of plant pathogenic bacteria of IAPAR (Instituto Agronômico do Paraná, PR, Brazil). This strain was grown at 28°C in Nutrient Broth (Difco™ NB - 5 g/L peptone and 3 g/L meat extract) or Tryptone Sucrose Broth (TSE - 10 g/L tryptone, 10 g/L sucrose and 1 g/L sodium glutamate) media with shaking at 200 rpm for 16 hours. The cells were collected at an optical density (OD_600_) corresponding to the log phase.

The extraction of total proteins was performed as described by Mehta & Rosato [17]. The cells were collected by centrifugation, and the pellet was washed in phosphate buffer (1.24 g/L K_2_HPO_4_; 0.39 g/L KH_2_PO_4_; 8.8 g/L NaCl, pH 7.2). Next, the pellet was suspended in 0.75 mL of extraction buffer (0.7 M sucrose; 0.5 M Tris-HCl, pH 7; 30 mM HCl; 50 mM EDTA; 0.1 M KCl and 40 mM DTT) and incubated for 15 min at room temperature. The same volume of phenol was added; after 15 min of agitation, the suspension was centrifuged at 10000 *g *and 4°C for 3 min, and the supernatant was recovered. This procedure was repeated twice.

The proteins were precipitated with five volumes of 0.1 M ammonium acetate in methanol, and the precipitate was washed once with 80% acetone. Protein concentrations were estimated by the Bradford method (Bio-Rad, Hercules, CA, USA). A lysate sample containing 1 mg of protein was denatured by adding 50 μL of buffer (8 M urea and 25 mM NH_4_HCO_3_, pH 8.0), reduced with 10 mM DTT at 37°C for 1 h, and alkylated with 50 mM iodoacetamide in the dark for 30 min. The urea concentration was reduced to 1 M by dilution. A 20-μg sample of trypsin (Promega, modified sequencing grade) (1:50) was added to digest the proteins at 37°C overnight. Neat formic acid was added to stop the digestion.

### Strong cation exchange (SCX) chromatography

Tryptic peptides were fractionated by strong cation exchange chromatography. The separation was performed on an SP-Sepharose HP™ column (GE Healthcare) using the AKTA PRIME™ chromatography system (GE Healthcare). The column was washed with buffer A (25 mM NH_4_HCO_3_, pH 3.0), and the peptides were eluted with buffer B (25 mM NH_4_HCO_3_, 500 mM KCl, pH 3.0) at a flow rate of 1 mL per minute. Buffer B concentrations of 10%, 20%, 25% and 30% were used to displace fractions during SCX chromatography. Fractions (about 100) were collected at 1 min intervals and concentrated by vacuum centrifugation to produce a final volume of 100 μL.

### The nLC-MS/MS System

The SCX fractions were loaded into a Waters CapLC™ system (Waters, Milford, MA). The digested proteins were desalted in-line using a Waters Opti-Pak C18 trap column. The sample injection volume was typically 10 μL, and the LC was performed using a NanoEase C18™ 150 mm × 75 μm column (Waters, Milford, MA) with elution (0.6 μL/min) using a linear gradient (10 to 50%) of acetonitrile containing 0.1% formic acid (solution B).

Electrospray tandem mass spectra were recorded using a Q-Tof quadrupole/orthogonal acceleration time of flight spectrometer (Waters, Milford, MA) interfaced with the CapLC™ capillary chromatograph. The ESI voltage was set at 3500 V using a metal needle, the source temperature was 100 ºC and the cone voltage was 100 V. The instrument control and data acquisition were conducted by a MassLynx data system (Version 4.0, Waters) and experiments were performed by scanning from a mass-to-charge ratio (m/z) of 200 to 2000 using a scan time of 1 s applied during the entire chromatographic process. The mass spectra corresponding to each signal from the Total Ion Current (TIC) chromatogram were averaged, allowing an accurate molecular mass determination. Exact mass MS/MS is achieved automatically using the Q-Tof's LockSpray™ (Waters, Milford, MA). The reference ion used was the mono charged ion of Rifampicin at m/z 823.4130.

### Database Searching

All data were processed using the ProteinLynx Global server (version 2.0, Waters). Proteins were identified by correlation of tandem mass spectra and *Xanthomonas axonopodis *pv. *citri *str. 306 genome data bank available at NCBI, using the MASCOT™ software (Matrix Science, version 2.1). One missed cleavage per peptide was allowed and an initial mass tolerance of 0.05 Da was used in all searches. Cysteines were assumed to be carbamidomethylated and variable modification of methionine (oxidation) was allowed. To evaluate the false positive rate of this approach, a reversed sequence databank (a database in which the sequences have been reversed) containing the same number of proteins in the *Xac *database was constructed.

## Abbreviations

Xac: *Xanthomonas citri *subsp. *citri*; **nLC**: capillary liquid chromatography; **nLC-MS/MS**: capillary liquid chromatography/tandem mass spectrometry; **SCX**: strong cation exchange chromatography; **2D LC-MS/MS**: two-dimensional liquid chromatography/tandem mass spectrometry; **NB**: Nutrient Broth; **TSE**: Tryptone Sucrose broth enriched with glutamic acid.

## Competing interests

The authors declare that they have no competing interests.

## Authors' contributions

MRS, APF and RMF carried out the proteomics experiments. MRS, LMM and FCG were responsible for bioinformatic analysis and have been involved in drafting the manuscript. FCG has made substantial contributions to analysis and interpretation of data. RM, JCFO, MIT and JAF made substantial contributions to the study conception and design and critically revised the manuscript for intellectual content. All authors edited the manuscript and approved the final version.

## Supplementary Material

Additional file 1 **Strong cation exchange chromatography**. Tryptic peptides were fractionated by strong cation exchange chromatography. Fractions (about 100) were collected at 1 min intervals and concentrated by vacuum centrifugation to produce a final volume of 100 μL. Figure A shows the chromatogram of peptides from *Xac *proteins detected in TSE medium and Figure B shows the chromatogram of peptides from *Xac *proteins detected in NB medium. Click here for file

Additional file 2 **Proteins identified of *Xanthomonas citri *subsp. *citri***. Complete list of all proteins expressed in both conditions available, categorized according annotation. Click here for file

Additional file 3 **Map of identified proteins in TSE × NB conditions**. The comparison between expressed products of *Xac *show more regions of continuous genes in TSE medium, showing that NB expressed minor number of proteins. The red color represents *Xac *proteome in TSE, yellow color represents *Xac *proteome in NB, black represents protein detected in both media and white refers to proteins not detected. Gray boxes represent hypothetical or conserved hypothetical proteins and green boxes show the proteins expressed only in TSE medium Click here for file

Additional file 4 **Functions assigned to some hypothetical proteins**. A BLAST search for homologous proteins in *Xanthomonas campestris *pv. *vesicatoria *returned 209 proteins with identity > 34%, coverage > 49% and e-values less than or equal to 10^-19^. Click here for file

Additional file 5 **List of proteins related to pathogenicity or virulence exclusively detected in TSE medium**. Click here for file
