# Supplementary material for: Proteome of the phytopathogen Xanthomonas citri subsp. citri: a global expression profile
Source: Proteome Sci. 2010 Nov 9;8:55. doi: 10.1186/1477-5956-8-55 (PMC2996358; doi:10.1186/1477-5956-8-55)
Supplement: Additional file 1 — Strong cation exchange chromatography. Tryptic peptides were fractionated by strong cation exchange chromatography. Fractions (about 100) were collected at 1 min intervals and concentrated by vacuum centrifugation to produce a final volume of 100 μL. Figure A shows the chromatogram of peptides from Xac proteins detected in TSE medium and Figure B shows the chromatogram of peptides from Xac proteins detected in NB medium. [file 1477-5956-8-55-S1.DOC]

## TSE medium
