# Supplementary figures and images for: Proteome of the phytopathogen Xanthomonas citri subsp. citri: a global expression profile
Source: Proteome Sci. 2010 Nov 9;8:55. doi: 10.1186/1477-5956-8-55 (PMC2996358; doi:10.1186/1477-5956-8-55)

**Map of identified proteins in TSE x NB conditions**


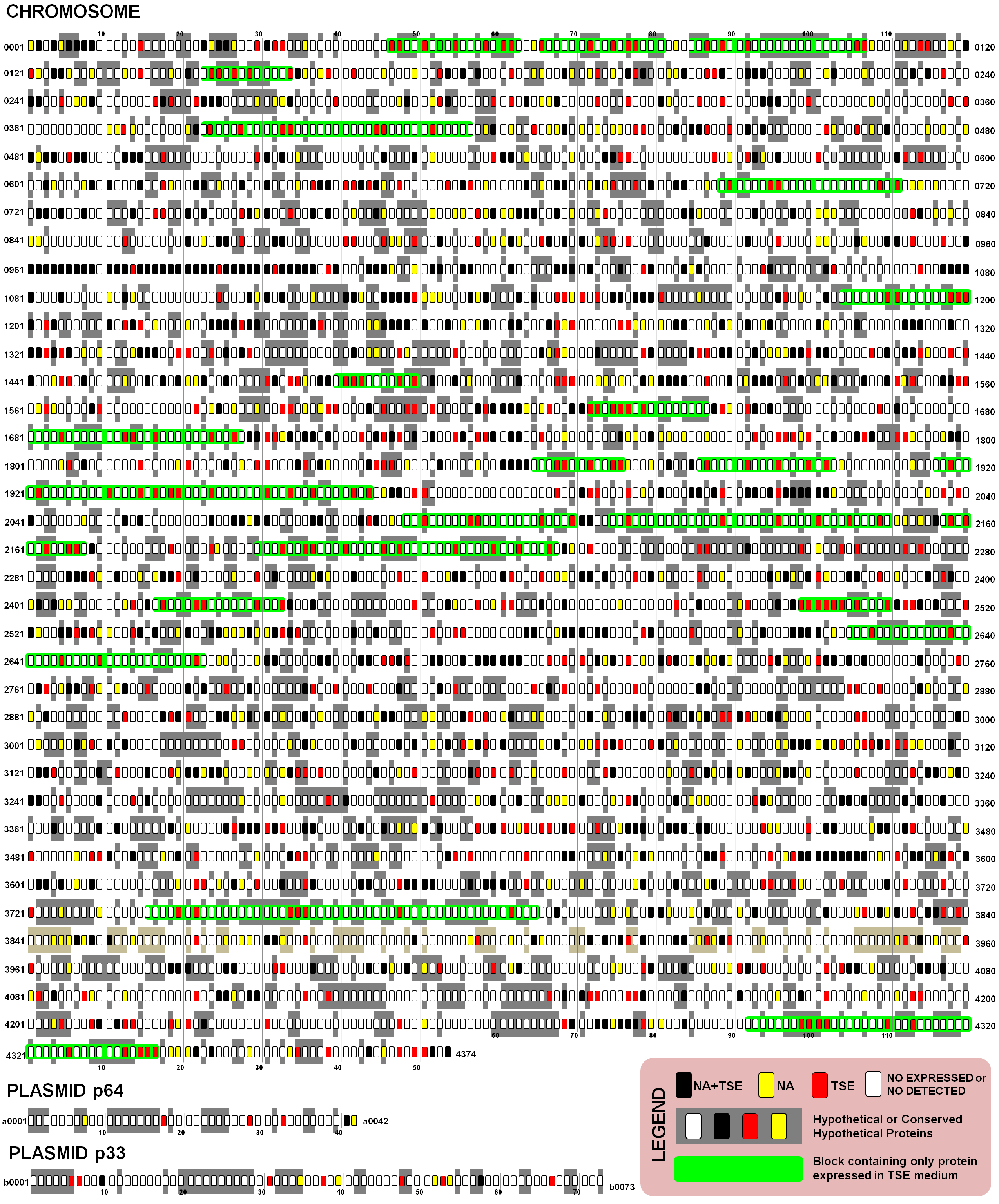

Supplement: Additional file 3 — Map of identified proteins in TSE × NB conditions. The comparison between expressed products of Xac show more regions of continuous genes in TSE medium, showing that NB expressed minor number of proteins. The red color represents Xac proteome in TSE, yellow color represents Xac proteome in NB, black represents protein detected in both media and white refers to proteins not detected. Gray boxes represent hypothetical or conserved hypothetical proteins and green boxes show the proteins expressed only in TSE medium [file 1477-5956-8-55-S3.DOC]
