# Supplementary material for: Proteome of the phytopathogen Xanthomonas citri subsp. citri: a global expression profile
Source: Proteome Sci. 2010 Nov 9;8:55. doi: 10.1186/1477-5956-8-55 (PMC2996358; doi:10.1186/1477-5956-8-55)
Supplement: Additional file 5 — List of proteins related to pathogenicity or virulence exclusively detected in TSE medium. [file 1477-5956-8-55-S5.DOC]

##### List of proteins related to pathogenicity or virulence exclusively detected in TSE medium.

| TSE |  |
| --- | --- |
| Gene_ID | Product |
| XAC0030 | Cellulase |
| XAC0393 | HpaF protein |
| XAC0394 | HrpF protein |
| XAC0405 | HrcV protein |
| XAC0406 | HrcU protein |
| XAC0412 | HrcN protein |
| XAC1368 | virulence protein |
| XAC1815 | filamentous haemagglutinin |
| XAC1918 | hemolysin related protein |
| XAC2197 | hemolysin- type calcium binding protein |
| XAC2201 | hemolysin secretion protein D |
| XAC2986 | pectate lyase II |
| XAC3516 | cellulase |
| XAC0313 | type V secretory pathway protein |
